# The blemishes of modern society? Acne prevalence in the Dogon of Mali

**DOI:** 10.1093/emph/eow027

**Published:** 2016-09-20

**Authors:** Christine E. Campbell, Beverly I. Strassmann

**Affiliations:** ^1^Department of Anthropology & Institute for Social Research, University of Michigan, Ann Arbor, MI, USA

## Abstract

**Background and Objectives:** Non-communicable diseases may reflect an evolutionary mismatch between our human ancestry and modern environments. To explore the mismatch hypothesis for *Acne vulgaris*, we studied the prevalence and severity of acne in Dogon adolescents in Mali, West Africa.

**Methodology:** We graded the prevalence and severity of acne in 1182 Dogon adolescents aged 11–18 years from nine villages using facial photos taken as part of a prospective cohort study. Eighty-nine (89%) of the individuals in the cohort migrated to the city during adolescence, enabling us to assess the effect of urban migration. Using multivariable logistic regression, we estimated the effect of predictor variables on the presence of acne.

**Results:** The prevalence of acne in the cohort was 28%, with 90% of cases being mild or very mild. Thus, the prevalence and severity of acne was much lower than for adolescents in high-income countries. Controlling for age, puberty, and body mass index (BMI), the odds of boys developing acne was 85% lower in the city than in the villages (*P* = 0.002).

**Conclusion and Implications:** Acne is similar to the ‘diseases of civilization’ in being promoted by the pro-inflammatory properties of modern diets. The low prevalence and severity of acne in the Dogon supports the mismatch hypothesis and suggests that acne should join the list of diseases of modern lifestyles. However, we also observed an unexpected decrease in acne in urban boys. Future research is needed for a deeper mechanistic understanding of the interplay between diet, inflammation, immune function and other environmental exposures that differ between urban and rural environments.

## Abstract (precis)

We conducted the first large sample, multivariable study of acne among adolescents in a traditional population. The low prevalence and severity of acne supports the hypothesis that acne should join the list of “diseases of civilization.” However, our results also showed a puzzling decrease in acne in urban boys.

## INTRODUCTION

In high-income countries, *Acne vulgaris* is one of the most common skin diseases [1–5] and one of the most frequent diagnoses for patients who visit a dermatologist [6]. Although not life threatening, the lesions and scarring of *Acne vulgaris* can cause significant perception of disfigurement [3,7], decreased self-esteem [7], depression [8] and lower prospects for employment [9]. *Acne vulgaris* is a multifactorial disease [10,11] that is influenced by four agreed upon factors: (1) hyperkeratinization and obstruction of sebaceous follicles caused by abnormal desquamation of the follicular epithelium; (2) androgen-stimulated increase in sebum production, (3) colonization of hair follicles in the skin by *Propionibacterium acnes*, and (4) inflammation [4,5,12–14]. The pathogenesis of acne is similar across different ethnic populations [6] and acne has been documented within all Fitzpatrick skin types (The Fitzpatrick scale is a numerical classification of skin color from I to VI with Type I being the fairest (albino, very fair with freckles) to Type VI being the darkest (deeply pigmented, ′black′ skin)) [6,15]. Here we consider *Acne vulgaris* within the framework of evolutionary medicine.

Human bodies are imperfectly adapted for a variety of reasons including the ‘mismatch’ between our genes and novel features of the environment [16]. However, there was no singular Environment of Evolutionary Adaptedness or specific time-period of our evolutionary past to which humans are adapted [17–19]. Instead different aspects of human biology evolved in different time periods [19,20]. The early emphasis on Stone Age genes was unwarranted [21] as many genes are far older (e.g. Hox genes) and others are more recent than the Pleistocene (e.g. genes for lactase persistence) [20,22]. Nor was the advent of agriculture the only disease-inducing transition in the human past [19]. Environmental and lifestyle changes relevant to disease risk occurred both long before and long after the end of the Stone Age [19].

Although it is helpful to put the Stone Age in perspective, discarding the mismatch hypothesis is unjustifiable. When the environment changes, traits that were adaptive in the previous environment become maladaptive in the new environment until natural selection has ‘caught up’. Quite simply, natural selection takes time to act [23–25]. A striking example was proposed by Janzen and Martin (1982) [26], who studied the peculiarly large and hard fruits of many trees in the tropical forests of Central America. These fruits appear to be anachronistically adapted for dispersal by gomphotheres (a relative of the mastodon with powerful molar teeth) and other large mammals that went extinct about 10 000 years ago. Ten millennia has not been long enough for the evolution of fruits that will be eaten and dispersed by smaller mammals, so these overly-large and overly-protected fruits rot under their parent trees [24,26]. Similarly, our study of acne is predicated on the understanding that after the environment changes, it will take time for natural selection to catch up.

Here we report the prevalence and severity of acne in Dogon adolescents in Mali, West Africa. We hypothesized a low prevalence and severity of acne in the Dogon on account of their traditional life-style, including a diet based on millet farming with limited consumption of novel, pro-inflammatory, fast foods. Inflammation plays an important role in the development of acne [27] and in the aetiology of other chronic diseases [28] associated with modern lifestyles. Examples include cardiovascular disease [29,30] type 2 diabetes mellitus [29], Alzheimer’s disease [31], and various cancers [32]. Modern dietary habits promote inflammation, especially consumption of foods that have a high glycemic load or a high ratio of omega-6 to omega-3 polyunsaturated fatty acids (PUFAs) [28]. The evidence that pro-inflammatory foods simultaneously exacerbate acne [4] and other chronic diseases [28], raises the possibility that *Acne vulgaris* (especially inflammatory acne) should be added to the list of ‘diseases of civilization’.

We also hypothesized that acne in the Dogon is aggravated by urban migration—a globally important source of environmental novelty in the 21^st^ century. The world’s population is >50% urban and will be 66% urban by 2050 [33]. The Dogon are migrating to Bamako, which is the fastest growing city in Africa and the sixth fastest growing city in the world [34]. When rural adolescents from farming families move to the city, they are introduced to a radically different environment compared to the one they experienced during development. In the city, one might expect a more acnegenic diet due to increased consumption of pro-inflammatory foods such as red meat [35] milk [36], food with a high glycemic index [12, 37–40], or a high ratio of omega-6 to omega-3 fatty acids [42–46]. One might also expect increased psychosocial stress due to loss of kin support or increased competition from wealthy urbanites. Further, in the city people tend to be more sedentary and do not get the stress-reducing benefits of aerobic farm work [47].

Previous studies reported a low prevalence of acne in traditional populations. For example, the prevalence of acne was 0% in the Kitavan Islanders of Papua New Guinea and the Aché foragers of Paraguay [4], 6.9% in adolescents of a roadside Ethiopian community [48], and 4.2% in adolescents in a cross-sectional, hospital-based study in Mali [49]. Furthermore, several studies reported that the Inuit [50] and Chinese [51] only developed acne after transitioning to a modern lifestyle. However, these studies [4, 48] had very small sample sizes of adolescents: from 47 to 300 individuals (Table 1). Our study is the largest (*N* = 1182) study of adolescents in a traditional, rural population and it has the advantage of being population rather than hospital or school-based [52]. Whereas previous studies controlled for few covariates—at most age or age and body mass index (BMI) [53,54], we explored multivariable models in which we tested the simultaneous effects of age, BMI, puberty, wealth and urban versus rural living on presence or absence of acne. Furthermore, our study design is unique in that we compared urban and rural members of the same cohort from nine source villages, thereby reducing other causes of heterogeneity between individuals. The members of our study population had the same surname, reducing genetic variability, and since they came from villages that were within 5 km of each other, cultural variability was minimal.

**Table 1.** Studies of adolescent acne prevalence by country

Background of Community Studied	Country	Age (years)	N	Total Prevalence (%)	Moderate to Severe (% of Total Prevalence)	Reference^b^
Subsistence horticulture, fisherman	Papua New Guinea	15–25	300	**0**	**0**	Cordain et al. 2002
Roadside town of 4000, subsistence farming	Ethiopia	10–16	47	**2.7**	NR^a^	Figueroa et al. 1996
Millet farming, rural villages, & city of 2 million	Mali	11–18	1182	**28.4**	9.2	This study
10 rural villages of Kakamega county	Kenya	10–19	133	**38.3**	N/A	Kiprono et al. 2015
Students in town of Arequipa, population 800,000	Peru	12–18	2214	**41.7**	19.0	Freye et al.1998
Secondary school in Nottingham, population 310,000	United Kingdom	14–16	317	**50.0**	27.4	Smithard et al. 2001
Eskisehir province (developed) and surroundings (rural)	Turkey	13–18	2230	**60.0**	21.4	Aksu et al. 2011
Randomly selected secondary schools	New Zealand	12–18	9398	**67.3**	20.9	Purvis et al. 2004
Secondary schools in state of Victoria	Australia	13–18	797	**83.5**	21.0	Kilkenny et al. 1998
Secondary schools and colleges	Singapore	13–19	1045	**88.0**	48.6	Tan et al. 2007
Schools in Kaduna, population 1.5 million	Nigeria	11–19	418	**90.7**	7.3	Yahya 2009
Students in Tehran, population 8 million	Iran	12–20	1,002	**93.2**	15.0	Ghodsi et al. 2009
Students in Antwerp, population 500,000	Belgium	14–18	594	**95.0**	38.0	Nijsten et al. 2005

a NR= no results.

b Grading scale information: (1) Cordain 2002: 3 grades: mild, moderate, severe; (2) Figuerora et al. 1996: presence of acne ‘Yes’ or ‘No’; (3) This study: 5 point scale (see Table 1); (4) Kiprono et al. 2015: not available; (5) Freye et al. 1998: 4 point scale: minimal, mild, moderate severe*; (6) Smithard et al. 2001: 3 grades: very mild, mild, moderate to severe; (7) Aksu et al. 2011: 4 grades: minimal, mild, moderate, severe*; (8) Purvis et al. 2004: 3 grades: None, Some, Severe; (9) Kilkenny et al. 1998: 4 point scale: minimal, mild, moderate, severe; (10) Tan et al. 2007: 3 grades: mild, moderate, severe; (11) Nijsten et al. 2005: 3 grades: little, moderate, severe; (12) Yahya 2009: GAGS 30 point scale*; (13) Ghodsi et al. 2009: 3 grades: mild, moderate, severe; *refer to paper for specific criteria.

### Ethnographic background

The Dogon villages we studied are on the Bandiagara Escarpment in central Mali and retain some of the salient features of the human evolutionary past including the absence of contraception, high fertility and mortality, polygyny and life in a tight-knit web of kin [52,55]. The Dogon are farmers whose main subsistence crop is pearl millet (*Pennisetum glaucum*), an ancient West African grain [56] that is usually eaten twice per day. Other local cereals include sorghum (*Sorghum bicolor*), fonio (*Digitaria exilis*) and rice (*Oryza glaberrima*) [57]. Mangos, oranges, bananas, papayas, guavas, tomatoes and wild fruits are part of the diet; whereas vegetables, such as eggplant, play a minor role with the exception of onions–the primary cash crop. Dairy products are rarely consumed although canned or powdered milk is added to Nescafé, a luxury item. Locally grown peanuts are commonly eaten as snacks and beans are another source of protein. In wealthier families, adult men consume morsels of fresh meat (beef, mutton, goat, pork or chicken). Refined sugar is added to porridge and beverages like wild fruit drinks, Nescafé, Lipton and Arabic tea. Palm oil often substitutes for the traditional oil from the shea plant (*Vitellaria paradoxa*). Aside from palm oil and the small amount of meat, dairy, rice and refined sugar in the Dogon diet, most foods are non-inflammatory and have a low glycemic load. Thus, Dogon food stuffs contrast with the pro-inflammatory, processed foods in modern diets (e.g. white bread, soda, processed snacks) [58] (See SI Discussion).

## METHODOLOGY

The subjects are participants in a prospective cohort study initiated by BIS in 1998. In May 1998 all children age 0–5 years in 9 villages were enrolled in the study and, during the following two years, all newborns were added. Thus, the data are for the total population of children (*N* = 1698) in the specified age range in the 9 villages. By 2012, 245 children had died (14.5%), 1427 were alive (84.2%), and 23 (1.4%) were lost to follow-up. Among the living children, 127 had moved to the capital of Mali, Bamako, and a similar number had moved to other cities, including Abidjan, in Cote d’Ivoire. In the present study, we examined the facial identity photographs taken between November 2011 and April 2012 for 1263 adolescents (88% of the survivors), including 89 adolescents who had moved to the capital city of Bamako. A total of 1182 facial photos, or 94% of the total, were of sufficient quality to be graded for acne. These 1182 adolescents ranged in age from 11.5 to 18.8 years (mean age 14.8 ± 2.0) and consisted of 554 females and 628 males (46.9% and 53.1%, respectively). A total of 1093 of our subjects lived in the rural villages whereas 42 boys and 48 girls were living in the city at the time of evaluation. The images were enlarged and viewed via Windows Photo Viewer by CEC.

The grading system consisted of a 5-point scale developed by Dréno *et al.* (2011) [59] (Table 2). We chose this scale because of its wide range of grades (very mild to very severe), which improved the precision of classification. The high amount of melanin in the skin of these adolescents made an assessment of the level of inflammatory lesions difficult. Thus the major determinant for acne grade was the facial surface area with acne lesions, though we did note the presence of inflammatory lesions.

**Table 2.** Definition of acne grade and severity. Source: Dréno et al. (2011). Scale for determination of acne severity in Dogon adolescents

Grade	Severity	Description
0	Clear, No lesions	Residual pigmentation and erythema may be seen.
1	Very Mild	A few scattered open or closed comedones and very few papules.
2	Mild	Easily recognizable: *less than half the face involved.* A few open or closed comedones and/or a few papules and pustules
3	Moderate	More than half the face involved. Many open or closed comedones and/or many papules and pustules.
4	Severe	Entire face involved, covered with many papules and pustules and/or many open or closed comedones and rare nodules.
5	Very Severe	Highly inflammatory acne covering the face with presence of nodules.

Acne lesions and acne severity are generally divided into two categories: non-inflammatory and inflammatory. Non-inflammatory lesions are small 1–2 mm comedones, commonly known as white or black heads, whereas inflammatory lesions are 5–6 mm papules, pustules, or nodules [2,3]. Likewise, non-inflammatory acne, also known as comedonal acne, consists of smaller, less severe lesions [2,3], and inflammatory acne consists of larger, more inflamed lesions. Despite the differentiation between non-inflammatory and inflammatory lesions, some dermatologists attest that all acne lesions are inflamed to varying degrees [27].

In our study, evidence of at least one non-inflammatory lesion (comedone) qualified as ‘very mild’ acne (grade 1). Other grading systems do not consider the presence of one non-inflammatory lesion to be acne [2,60]. However, since comedones are often precursors to more severe acne and are evidence for some level of acne within the population, we considered it preferable to use a grading system that was conservative. Thus, our methodology classifies more individuals as having *Acne vulgaris* than might be the case for other grading schemas [2,60–62]. Due to the difficulty of accurately detecting and diagnosing scars and hyperpigmentation caused by acne, we did not consider these two factors in our classification. We created a binary acne variable, defined as follows: 0 = absent, 1 = present (any grade) because of the high prevalence of no acne.

A female Dogon research assistant visually inspected the girls’ breasts without palpation and scored them according to Tanner breast stage [63]. A girl was considered to be pubertal if she had reached at least stage 2 as this breast stage is defined as the onset of thelarche (breast budding) and is the marker for puberty [64]. For girls, we gathered data on menarche (status quo) and buttocks circumference (cm), and for boys, we assayed saliva samples to measure testosterone levels (pg/ml). Each subject’s height (cm), weight (kg), BMI (kg/m^2^), percent body fat, abdominal skinfold thickness (mm), subscapular skinfold thickness (mm), waist circumference (cm) and wealth rank (z-score) was measured. Location of residence (urban or rural) was also recorded.

Approval for the fieldwork was obtained from the Malian government and the University of Michigan Health Sciences and Behavioural Sciences Institutional (HSBS) Review Board (HUM00013631). Permission to score the existing photos for acne in a way that protected anonymity was determined to be exempt from IRB regulation (HUM00076491) on 5/10/2013 by the HSBS IRB at the University of Michigan.

### Statistical methods

We ran Spearman rank correlations [65] between our binary acne variable and each of our candidate predictor variables. We then created multivariable logistic regression models, in which the dependent variable was the presence or absence of acne and the predictors were age, BMI, pubertal development, wealth and urban vs. rural living (Table 3). We analyzed the data separately for each sex as several variables were pertinent only to girls (breast stage, menarche and buttocks circumference) or boys (e.g., testosterone). All analyses were carried out using IBM SPSS (Statistical Package for the Social Sciences) software (versions 18–22) [66]. We considered the results to be statistically significant if *P* < 0.05.

**Table 3.** Multivariable logistic regression results with acne (0 = not present, 1 = present) as the dependent variable; and age, BMI, puberty, wealth and urban versus rural residence as predictor variables

Model 1	Girls			Boys		
	**Exp (B)**	**95% C.I.**	**P**	**Exp (B)**	**95% C.I.**	**P**
Age (years)	1.253	1.057–1.485	**0.009**	1.426	1.205–1.688	**0.000**
BMI (kg/m^2^)	1.129	1.017–1.253	**0.023**	1.323	1.119–1.565	**0.001**
Puberty (Girls: 0 = no, 1 = yes; Boys: Testosterone (pg/ml))	2.187	1.144–4.184	**0.018**	1.013	1.004–1.023	**0.006**
Wealth (z-score)	0.933	0.736–1.183	**0.566**	0.992	0.762–1.291	**0.951**
Urban living (no, yes)	0.578	0.208–1.608	**0.293**	0.148	0.044–0.505	**0.002**

## RESULTS

### Acne severity

**Figure 1.**
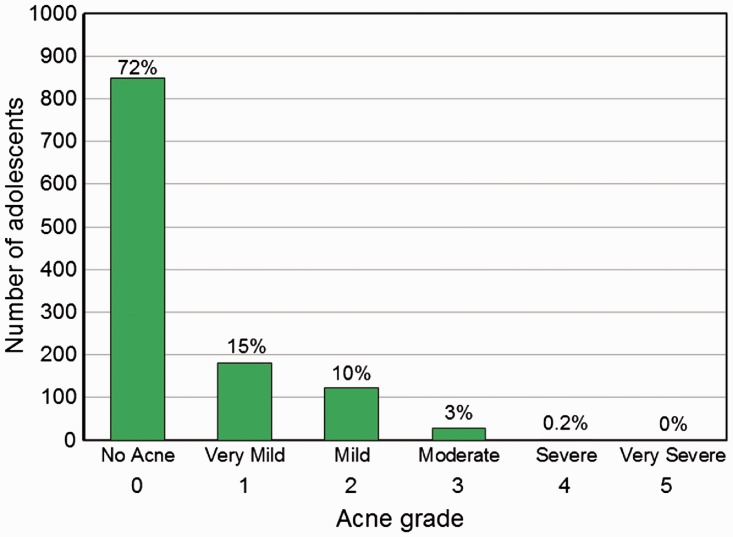
Number of Dogon adolescents (age 11–18) with each acne grade (percentage of population above bar)

**Figure 2.**
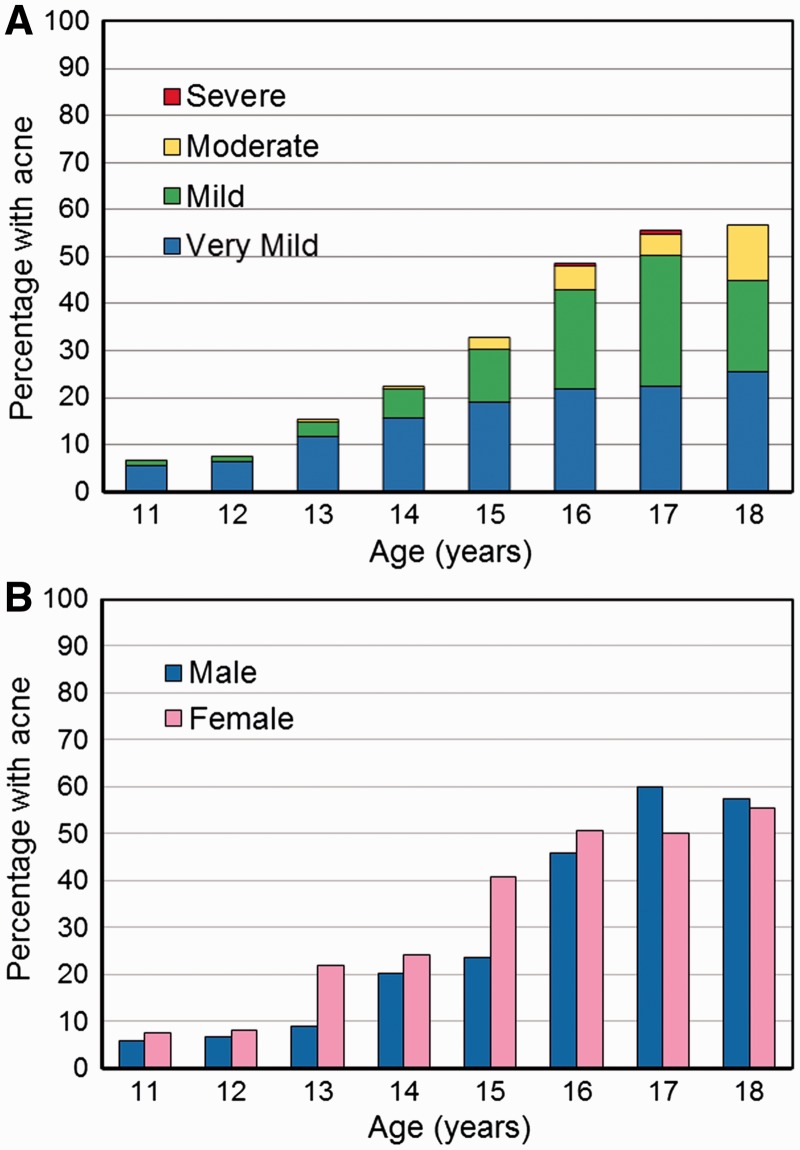
Percentage of population with acne (any grade). (**A**) by severity and age (years); (**B**) by sex and age (years)

The prevalence of acne by grade for the 1182 Dogon adolescents was as follows: no acne: 71.8% (*N* = 849), very mild: 15.3% (*N* = 182), mild: 10.4% (*N* = 123), moderate: 2.5% (*N* = 29), severe: 0.2% (*N* = 2) and very severe: 0% (*N* = 0) (Fig. 1). Cases of moderate acne did not occur until age 13 (3.1% of all 13 year olds) and increased to 11.9% by age 18 years (Fig. 2A, Supplementary Material, Table S1).

### Acne prevalence

Using our binary acne variable (0 = absent, 1 = present), the presence of acne increased progressively for the entire cohort from 6.6% at age 11 to 56.7% at age 18 (Supplementary Material, Table S1). The overall prevalence across all ages was 26.4% in males and 30.5% in females (Supplementary Material, Table S1). The distribution by age was similar for boys and girls, although the increase with age occurred earlier in girls (Fig. 2B; Supplementary Material, Table S1).

**Figure 3.**
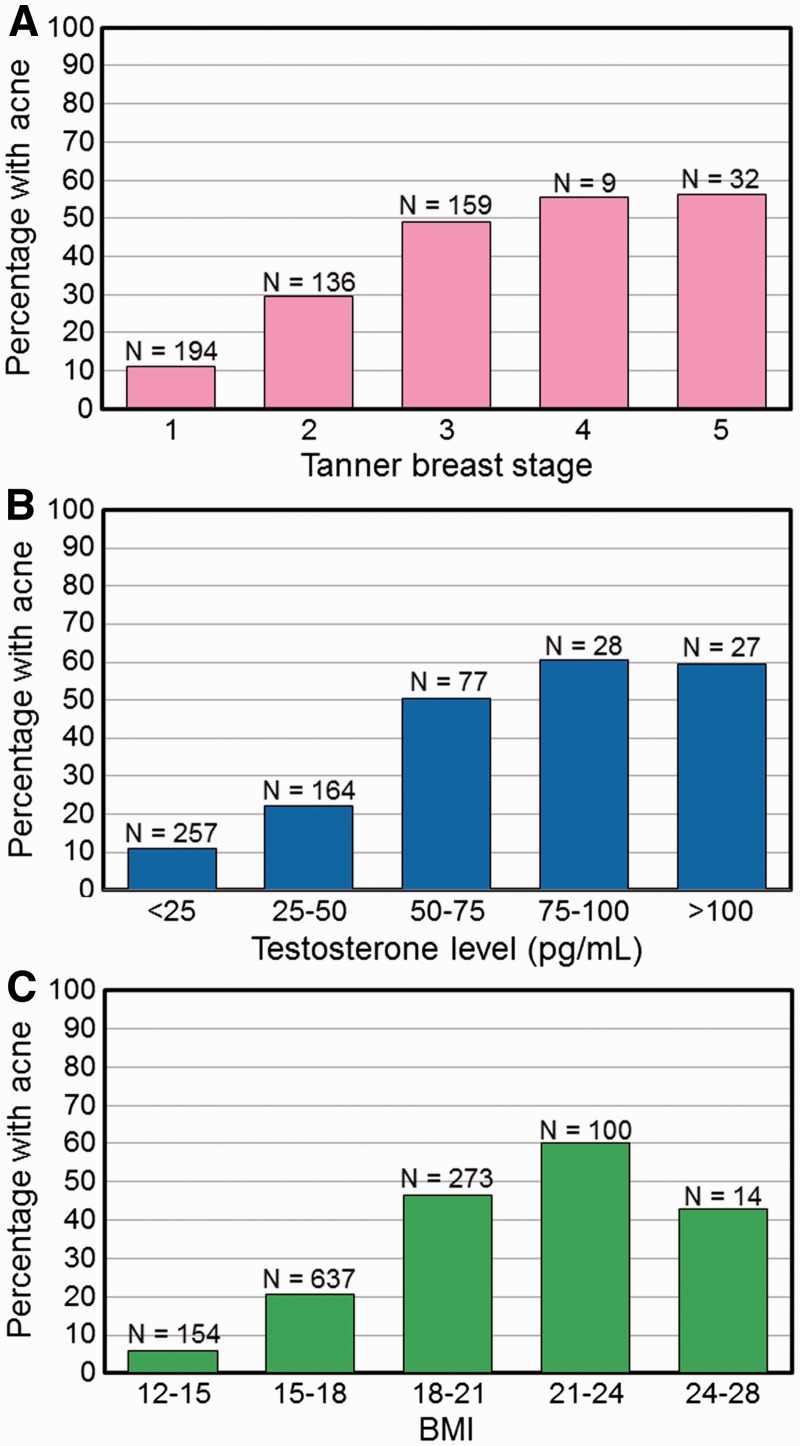
Percentage of population with acne (any grade). (**A**) by Tanner breast stage (1 - 5); (**B**) by testosterone level (pg/ml); (**C**) by BMI (kg/m^2^)

Acne (any severity) was present in 23.6% of girls who had not reached menarche and in 51.1% who had reached menarche. The prevalence of acne increased with Tanner breast stage (Fig. 3A) in girls and with testosterone level in boys (Fig. 3B). Acne prevalence increased with BMI until BMI reached >25 kg/m^2^, after which the sample size was small (Fig. 3C). Among the individuals who had acne, 17.6% had at least one inflammatory lesion and 82.4% had no inflammatory lesions.

### Spearman rank correlation coefficients: bivariate results

In both sexes, acne presence (having any acne) was significantly positively correlated with age, BMI, height, weight, subscapular skinfold, abdominal skinfold and waist circumference. In girls, acne presence was additionally correlated with buttock circumference, tanner breast stage, and menarche. In boys, acne presence was correlated with testosterone level (pg/ml). Percent body fat was significantly negatively correlated with acne presence in boys and significantly positively correlated with acne in girls. Wealth (z-score) was not significantly correlated with acne prevalence for boys or girls (girls: Spearman’s ρ = 0.006, *P* =0.849; boys: Spearman’s ρ = 0.031, *P* =0.452) (Supplementary Material, Table S2A and B). In the bivariate results, living in an urban setting was weakly, positively correlated with acne presence for both boys and girls although not quite significantly so (girls: Spearman’s ρ = 0.075, *P* = 0.079; boys: Spearman’s ρ = 0.075, *P = *0.060) (Supplementary Material, Table S2A and B).

### Logistic regressions: multivariable results

The multivariable logistic regression results are shown in Table 3. Descriptive statistics for the variables in the model, broken down by gender (female/male) and residence (urban/rural), are shown in Table 4. In both sexes, the odds of having acne increased with age. Specifically, for a 1 year increase in age, the odds of having acne increased 25% in girls (*P* = 0.009) and 43% (*P* = 0.000) in boys. For a one unit (kg/m^2^) increase in BMI, the odds of acne increased 13% in girls (*P* = 0.023) and 32% (*P = * 0.001) in boys. For females, having a Tanner Breast stage of two or higher (indicative of having reached puberty) increased the odds of having acne by a factor of 2.2 (*P* = 0.018), and in males, a 10 pg/ml increase in testosterone increased the odds of having acne 13% (*P =*0.060). Wealth was not significantly related to acne presence in either sex. Urban living was not significantly associated with the acne presence in girls; however, in boys, moving to the capital city, Bamako, decreased the odds of having any acne by 85% (*P* = 0.002) (Table 3).

**Table 4.** Descriptive statistics (mean, standard deviation and sample size) by gender and rural versus urban residence; acne prevalence; and acne grade in Dogon adolescents.

	Female				Male			
	Rural		Urban		Rural		Urban	
*N*	506		48		586		42	
	Mean	SD	Mean	SD	Mean	SD	Mean	SD
Age (years)	**14.6**	1.85	**16.4**	1.99	**14.6**	1.97	**16.6**	1.96
BMI (kg/m^2^)	**17.5**	2.72	**21.3**	3.21	**16.9**	1.82	**18.8**	2.24
Puberty (Girls: 0 = no, 1 = yes; Boys: Testosterone (pg/ml))	**0.6**	0.49	**0.9**	0.32	**33.2**	25.20	**86.9**	55.19
Wealth (z-score)	**0.5**	0.90	**0.9**	0.90	**0.5**	0.92	**0.6**	0.60
**ACNE**	**%**	***N***	**%**	***N***	**%**	***N***	**%**	***N***
**Prevalence** (any grade)	29.4	149	41.7	20	25.6	150	38.0	16
	**Mean**	**SD**	**Mean**	**SD**	**Mean**	**SD**	**Mean**	**SD**
**Grade** (0–5)	0.5	0.81	0.7	0.99	0.4	0.74	0.7	0.93

## DISCUSSION

### Variation in acne prevalence and severity

Acne occurs in the Dogon adolescent population (age 11.5–18.8) at a fairly low prevalence (28.3%) and severity, with the majority of cases (90.8%) being ‘very mild’ or ‘mild’ (Fig. 1, Table 4). In addition, the prevalence would be nearly cut in half (to 13.1%) if the lowest acne grade (very mild) were not considered acne—consistent with the grading method for three previous studies [61,67,68]. The low severity of acne suggests that the majority of Dogon adolescents had non-inflammatory acne. Although detecting inflammatory lesions was difficult, only 17.6% of Dogon adolescents with acne of any grade had ≥ 1 inflammatory lesion. In addition, we observed no other severe form of acne, such as *Acne conglobata* [69,70]

The low prevalence of acne in the Dogon contrasts with the higher prevalence reported for high income countries or urban areas of middle income countries where lifestyles are presumably more modern. For example, the prevalence of acne in adolescents living in Singapore [62], Australia [61], United Kingdom [67], Belgium [71] and New Zealand [68] ranged from 50 to 95%, with 21 to 47% of individuals with acne having a grade of moderate to severe (Table 1). Acne prevalence in urban communities of middle-income countries such as Peru [72], Iran [1], Turkey [73] and Nigeria [74], ranged from 42% to 93%. Among the individuals who had acne in these countries, 7.3% to 21.4% had the moderate to severe grades (Table 1). Thus, acne grade was higher (more severe) in the high than in the middle income countries and both acne grade and prevalence were conspicuously low in the Dogon, as we originally hypothesized.

Despite the low prevalence of acne in Dogon adolescents overall, we note that the prevalence did reach 56.7% at age 18 years, a percentage similar to urban Peruvians (city: Arequipa) of the same age (59.4%) [72], but lower than the 93.3% prevalence observed at ages 16–18 years in Australia [61] and the 97.1% prevalence observed at ages 17–19 years in urban Nigeria [74]. Although 56.7% of Dogon adolescents had acne at age 18, the majority of these cases (88.1%) were very mild or mild. The only studies that found the prevalence of acne to be lower than that of the Dogon were conducted in traditional, non-urban populations [4,48,75] (Table 1).

Acne prevalence in African communities varies greatly: from 0.1% in rural Tanzania (unspecified ages) [76], to approximately 4% in rural Cameroon (ages 10–19) [75], and 38% in rural Kenya (ages 10–19) [77]. In urban populations, acne prevalence reached 65% in Durban, the second most populous urban area in South Africa [78], for individuals 13–24 years of age; and 97% for individuals aged 17–19 in urban Nigeria [74]. In a study that compared both rural and urban communities in Ghana, 0.2% of rural school children had acne (*N* = 527) compared to 12.9% (*N* = 489, *P*<0.001) in the capital, Accra [53]. The Dogon have a lower acne prevalence than the rural Kenyans but a higher prevalence than the rural populations of Tanzania, Cameroon and Ghana. Furthermore, the Dogon had less acne than did Africans in urban environments, except Accra.

### Causes of variation in acne prevalence & severity (see SI discussion)

Lifestyle factors may explain the low prevalence of acne in the Dogon, including a low glycemic load diet [4,12, 37–40] and limited milk consumption [36]. The Dogon diet consists of food with glycemic indices as low as 1.7 (peanuts), 6.8 (mangoes) and 16.8 (millet), which contrasts sharply with the glycemic indices found in the traditional western diet (i.e. 34.7 for white bread and 64.9 for refined sugar) [4,58,79]. Multiple randomized controlled trials have found that a high glycemic load diet can increase the risk for acne [37–39]. Nonetheless, other aspects of the Dogon lifestyle such as sweetening hot drinks with refined sugar, consuming palm oil and psychosocial stress may contribute to the acne that we did observe (see SI Discussion).

### Predictors of acne in the Dogon

Three inter-related variables, age (years), BMI (kg/m^2^) and puberty (Testosterone pg/ml for boys and Tanner Breast stage ≥2 for girls) were significantly and positively associated with the presence of acne in Dogon adolescents, even when included in the same statistical model (Table 3). The positive correlation with age is consistent with previous studies [72,80–82] and is probably mediated by androgenic hormones released during puberty [83]. The finding that puberty for both girls and boys was positively correlated with acne replicates previous studies [80–83]. Lastly, the positive and highly significant correlation between acne presence and BMI was expected since BMI increases during puberty [82]. Other studies have reported acne to be associated with a high BMI (overweight and obesity) for girls [54] however, none have had sufficient data to evaluate individual predictors of acne when controlling for others in a multivariable model—as we have done (Table 3).

The odds ratios of our findings with respect to puberty in boys and girls are not directly comparable to each other. We used a threshold measure for puberty in girls (breast stage two or higher) and a continuous variable for testosterone level (pg/ml) in boys. A one unit increase in testosterone (pg/ml) is much less than the difference between having breast stage I versus II or higher in girls.

### Wealth

Interestingly, we found that wealth was not significantly correlated with acne development. Our expectation had been that a richer diet in wealthier families would lead to an increase in acne. It is possible that wealthier adolescents consumed more sugar, milk and rice, which are potential risk factors for acne [4,12, 37–40] but were also better nourished. Thus, there may have been opposing forces: better nutrition and immune function in the wealthier families counterbalanced by eating more foods with a high glycemic index like sugar. It is also possible that there wasn’t enough variation in diet to observe a correlation between acne and wealth.

### Urban vs. rural

Another significant and unexpected finding is that urban living was associated with an 85% decrease (*P* = 0.002) in the odds of having any acne for boys. The underlying mechanisms are unclear. It is possible that urban boys had access to a more nutritious diet that included a greater variety of vegetables that provide vitamins that support the immune system. In ongoing research, we are examining the effect of gender and urban migration on C-reactive protein, which is a marker for inflammation [84,85].

It is unclear why there was no difference in the odds of having acne by rural versus urban residence for girls. The lifestyles of boys and girls in the city differ—with girls likely to work as maids and boys more likely to be enrolled in high school (lycées). However, when we added a variable for attending school (no, yes) to the multivariable model it was not statistically significant for either sex (*P* = 0.68 for boys and *P* = 0.57 for girls) and it did not affect the odds ratio or p-value for urban residence or the other covariates in the model. Specifically, the reduction of acne prevalence in urban boys was 86% (*P* = 0.004) after adjusting for school in the model in Table 3. In ongoing research, we are investigating the effect of urban migration and gender on stress as measured by blood pressure, cortisol levels and the global perceived stress scale [86]. It will be helpful to know how urban migration affects stress levels in both sexes.

Our finding of lower odds of acne in urban boys is consistent with two studies in Turkey and Portugal. In these studies, the prevalence of acne in semi-rural or low-income students was higher than in students living in an urban environment [73,87]. The authors of the Turkish study speculated that this difference was caused by increased access to specialized medical doctors, such as dermatologists, in the city [87]. This explanation is unlikely to apply to the Dogon who, regardless of urban or rural residence, rarely visit specialists.

We note that 42 boys (6.7%) lived in the city and 586 (93.3%) lived in the rural villages. Likewise, only 48 girls (8.7%) lived in the city compared to 506 (91.3%) in the villages (Table 4). Although our sample size for urban adolescents was limited, we had a strong effect size and a highly significant *P*-value for the urban-rural comparison in boys (Odds ratio = 0.85, *P* = 0.002).

The reduced odds of acne in urban Dogon boys did not show up in the Spearman rank correlation test. In this bivariate test, living in an urban setting was weakly positively correlated with acne for both boys and girls, although not quite significantly so (girls: Spearman’s ρ = 0.075, *P = *0.079; boys: Spearman’s ρ=.075, *P* =.06) (Supplementary Material, Table 2A and B). A likely reason for the positive correlation in bivariate analyses is that the subjects who moved to the city were older, on average, than the subjects who remained in the rural area (Table 4). Moreover, age at puberty was earlier for Dogon adolescents who moved to the city (Table 4). The decrease in acne risk for urban boys showed up only in our multivariable models in which age, BMI, puberty and wealth were controlled. Thus bivariate studies of acne prevalence in urban versus rural settings may be misleading. Importantly, the subjects we measured in the city were from the same rural villages as the subjects we measured in the rural areas, increasing the validity of our rural-urban comparison.

### Study limitations

In our study, the acne grader (CEC) was not a certified dermatologist, however advice on acne grading was provided by two practicing dermatologists (Gerard Plewig MD of the University of Munich, Germany and Lisa Maier MD, clinician at the University of Michigan Health System). The photos of each adolescent were not taken specifically for acne grading purposes and hence lighting conditions, though similar, were not identical. Due to the high amount of melanin in the subjects’ skin, accurate detection of comedones and inflammation was challenging. Finally, comparison of acne prevalence and severity in the Dogon to that of other populations was made more difficult by the multiplicity of grading scales employed (Table 1)–to date, there is no universally agreed upon grading scale for acne.

Furthermore, puberty in the Dogon occurs relatively late, with girls reaching menarche about five years later than in the United States. As the age range of our subjects was 11.5 to 18.8 years, we cannot report on acne prevalence after age 19 years. Other studies of adolescent acne in African populations, had age ranges similar to those in our study: 10–16 years in Ethiopia [48], 10–19 years in Kenya [77] and 11–19 years in Nigeria [74]. We note that the prevalence of acne may be slightly underestimated when members of a late-maturing population (like the Dogon and other African populations) are compared by age to members of early maturing populations in Australia or Europe.

## CONCLUSION AND IMPLICATIONS

In comparison to studies of adolescents living in high income countries or urban areas, the overall prevalence of *Acne vulgaris* in the Dogon was low and the majority of cases were mild or very mild (90%). These results are consistent with the hypothesis that acne is a disease that is aggravated by modern lifestyles. However, this hypothesis predicts that acne should be higher in urban than in rural environments. Using a carefully controlled study design in which we compared members of the same cohort who migrated to the city against those who stayed in their rural villages, boys in the city actually had *lower* odds of having acne. In contrast with previous studies, we controlled for BMI, puberty and wealth. These results suggest that a more fine-tuned examination of the interplay between health, immune function, diet, inflammation, stress and environmental exposures is needed to shed light on acne risk (SI Discussion). Dichotomies like traditional versus modern, or urban versus rural, mask the underlying variation in risk factors that contribute to the aetiology of acne.

## Supplementary Material

Supplementary Data
