# Supplementary material for: The blemishes of modern society? Acne prevalence in the Dogon of Mali
Source: Evol Med Public Health. 2016 Sep 20;2016(1):325–37. doi: 10.1093/emph/eow027 (PMC5046992; doi:10.1093/emph/eow027)
Supplement: Supplementary Data [file supp_2016_1_325__index.html]

The blemishes of modern society? — Supplementary Data 

# The blemishes of modern society?

## Supplementary Data

files

- Supplementary Data - pdf file
- Supplementary Data - docx file
