# Supplementary material for: The blemishes of modern society? Acne prevalence in the Dogon of Mali
Source: Evol Med Public Health. 2016 Sep 20;2016(1):325–37. doi: 10.1093/emph/eow027 (PMC5046992; doi:10.1093/emph/eow027)
Supplement: Supplementary Data [file supp_eow027_Supplementary.pdf]

SI Table 1. Percent of Dogon adolescents with acne (no, yes) and acne grade (very mild, mild, moderate, severe, very severe) by age and sex.

	Age (year)	Total		With Acne		**Acne Grade**									
						Very mild		Mild		Moderate		Severe		Very Severe	
		N	%	N	%	N	%	N	%	N	%	N	%	N	%
**Both Sexes Total**		**1182**	**100**	**335**	**28.3**	**181**	**15.3**	**123**	**10.4**	**29**	**2.5**	2	0.2	0	0.0
	11	91	7.7	6	**6.6**	5	5.5	1	1.1	0	0.0	0	0.0	0	0.0
	12	189	16.0	14	**7.4**	12	6.3	2	1.1	0	0.0	0	0.0	0	0.0
	13	196	16.6	30	**15.3**	23	11.7	6	3.1	1	0.5	0	0.0	0	0.0
	14	181	15.3	40	**22.1**	28	15.5	11	6.1	1	0.6	0	0.0	0	0.0
	15	153	12.9	50	**32.7**	29	19.0	17	11.1	4	2.6	0	0.0	0	0.0
	16	175	14.8	85	**48.6**	38	21.7	37	21.1	9	5.1	1	0.6	0	0.0
	17	130	11.0	72	**55.4**	29	22.3	36	27.7	6	4.6	1	0.8	0	0.0
	18	67	5.7	38	**56.7**	17	25.4	13	19.4	8	11.9	0	0.0	0	0.0
**Males Total**		**628**	**53.1**	**166**	**26.4**	**92**	**14.6**	**60**	**9.5**	**14**	**2.2**	**0**	**0.0**	**0**	**0.0**
	11	51	8.1	3	**5.9**	2	3.9	1	2.0	0	0.0	0	0.0	0	0.0
	12	103	16.4	7	**6.8**	7	6.8	0	0	0	0.0	0	0.0	0	0.0
	13	100	15.9	9	**9.0**	8	8.0	1	1.0	0	0.0	0	0.0	0	0.0
	14	94	15	19	**20.2**	14	14.9	4	4.3	1	1.1	0	0.0	0	0.0
	15	72	11.5	17	**23.6**	12	16.7	4	5.6	1	1.4	0	0.0	0	0.0
	16	98	15.6	46	**46.9**	21	21.4	20	20.4	5	5.1	0	0.0	0	0.0
	17	70	11.1	42	**60**	17	24.3	22	31.4	3	4.3	0	0.0	0	0.0
	18	40	6.4	23	**57.5**	11	27.5	8	20.0	4	10	0	0.0	0	0.0
**Female Total**		**554**	**46.9**	**169**	**30.5**	**89**	**16.1**	**63**	**11.4**	**15**	**2.7**	**2**	**2.2**	**0**	**0.0**
	11	40	7.2	3	**7.5**	3	7.5	0	0	0	0.0	0	0.0	0	0.0
	12	86	15.5	7	**8.1**	5	5.8	2	2.3	0	0.0	0	0.0	0	0.0
	13	96	17.3	21	**21.9**	15	15.6	5	5.2	1	1.0	0	0.0	0	0.0
	14	87	15.7	21	**24.1**	14	16.1	7	8.0	0	0.0	0	0.0	0	0.0
	15	81	14.6	33	**40.7**	17	21.0	13	16.0	3	3.7	0	0.0	0	0.0
	16	77	13.9	39	**50.6**	17	22.0	17	22.0	4	5.2	1	1.3	0	0.0
	17	60	10.8	30	**50.0**	12	20.0	14	23.3	3	5.0	1	1.6	0	0.0
	18	27	4.9	15	**55.5**	6	22.2	5	18.5	4	14.8	0	0.0	0	0.0

SI Table 2A. Spearman rank correlations for boys

	Acne (none, some)	Age (years)	Urban living (no, yes)	Wealth (z-score)	Testos-terone (pg/mL)	BMI (kg/m^2)	Height (cm)	Weight (kg)	% Body Fat	Subscap-ular skin fold (mm)	Abdomin-al skinfold (mm)	Waist circum. (cm)
**Acne (none, some)**	1.000	0.441***	0.075	0.031	0.384***	0.415***	0.487***	0.492***	-0.147***	0.364***	0.365***	0.455***
**Age (years)**		1.000	0.235***	0.037	0.602***	0.652***	0.805***	0.807***	-0.469***	0.539***	0.517***	0.718***
**Urban living (no, yes)**			1.000	0.036	0.297***	0.195***	0.235***	0.232***	-0.051***	0.311***	0.322***	0.213***
**Wealth (z-score)**				1.000	-0.004	0.123***	0.093*	0.113***	0.004	0.077	0.104**	0.117***
**Testos-terone (pg/mL)**					1.000	0.530***	0.564***	0.597***	-0.166***	0.447***	0.452***	0.578***
**BMI (kg/m^2)**						1.000	0.681***	0.869***	0.031***	0.637***	0.555***	0.825***
**Height (cm)**							1.000	0.949***	-0.296	0.599***	0.970***	0.857***
**Weight (kg)**								1.000	-0.187***	0.660***	0.631***	0.918***
**% Body Fat**									1.000	0.003	-0.051	-0.102**
**Subscap-ular skin-fold (mm)**										1.000	0.687***	0.633***
**Abdonim-al skin-fold (mm)**											1.000	0.615***
**Waist (cm)**												1.000

Significance: *P < .05, **P < .01, ***P < .001

SI Table 2B. Spearman rank correlations for girls

	Acne (none, some)	Age (years)	Urban (no, yes)	Wealth (z-score)	Puberty (no, yes)	Tanner breast (1-5)	Menarche (no, yes)	BMI (kg/m^2)	Height (cm)	Weight (cm)	Buttocks circum-ference (cm)	% Body Fat	Subscap-ular skinfold (mm)	Abdom-inal skinfold (mm)	Waist circum-frence (cm)
**Acne (none, some)**	1.000	0.359***	0.075	-0.006	0.320***	0.367***	0.271***	0.345***	0.335***	0.377***	0.389***	0.198***	0.336***	0.298***	0.343***
**Age (years)**		1.000	0.250***	0.036	0.682***	0.785***	0.654***	0.715***	0.726***	0.798***	0.782***	0.378***	0.668***	0.552***	0.759***
**Urban (no, yes)**			1.000	0.081	0.158***	0.235***	0.298***	0.308***	0.185***	0.296***	0.320***	0.280***	0.372***	0.348***	0.303***
**Wealth (z-score)**				1.000	0.016	0.049	0.112**	0.082	0.090*	0.096*	0.116**	0.098*	0.082	0.058	0.102*
**Puberty (no, yes)**					1.000	0.876***	0.456***	0.658***	0.672***	0.725***	0.720***	0.426***	0.654***	0.509***	0.676***
**Tanner breast (1-5)**						1.000	0.704***	0.785***	0.724***	0.833***	0.839***	0.54***	0.761***	0.587***	0.786***
**Menarche (no, yes)**							1.000	0.653***	0.533***	0.667***	0.682***	0.482***	0.619***	0.487***	0.643***
**BMI (kg/m^2)**								1.000	0.664***	0.929***	0.913***	0.771***	0.822***	0.687***	0.892***
**Height (cm)**									1.000	0.883***	0.831***	0.471***	0.656***	0.566***	0.776***
**Weight (kg)**										1.000	0.998***	0.697***	0.827***	0.692***	0.926***
**Buttocks circum-frence (cm)**											1.000	0.708***	0.847***	0.706***	0.909***
**% Body Fat**												1.000	0.665***	0.579***	0.687***
**Subscap-ular skin-fold (mm)**													1.000	0.748***	0.801***
**Abdominal skinfold (mm)**														1.000	0.673***
**Waist circum-frence (cm)**															1.000

Significance values *P < .05, **P < .01, ***P < .001
