# Supplementary material for: The blemishes of modern society? Acne prevalence in the Dogon of Mali
Source: Evol Med Public Health. 2016 Sep 20;2016(1):325–37. doi: 10.1093/emph/eow027 (PMC5046992; doi:10.1093/emph/eow027)
Supplement: Supplementary Data [file supp_eow027_Supplementary_Discussion_Campbell_Strassmann.docx]

SI DISCUSSION

**Causes of Variation in Acne Prevalence & Severity.**

The Dogon’s low severity of acne may be related to certain aspects of their lifestyle such as a diet with a low glycemic load [1-6], limited milk consumption [7], low consumption of oils with high omega-6 to omega-3 ratios [8] and possibly limited exposure to halogenic environmental pollutants [9]. Nonetheless, other aspects of their lifestyle such sugar in hot drinks and consumption of palm oil may promote acne.

**Hormones & Diet.** Acne development is influenced by hormones such as testosterone, progesterone, dehydroepiandrosterone sulfate (DHEAS), glucocorticoids, insulin, and insulin-like growth factors (IGF-1) [10] that can develop endogenously (i.e. during puberty) or affect the body through exogenous means (e.g., high glycemic load diet, milk, pollutants) [10,11]. For example, certain foods, such as highly processed sugars have been shown to alter hormonal interactions, consequentially influencing acne development [6,11]. Specifically, processed sugar can induce hyperinsulinemia and insulin resistance ([1,10,12,13] affecting androgen levels through the increase of insulin, IGF-1, and glucorticoids [11,14]. Androgens, specifically DHEAS [11], augment sebum production, part of the pathogenesis of acne ([13,15,16].

**Glycemic Load.** The Dogon’s low glycemic load may explain their lower prevalence of acne. The glycemic load (GL) is a measurement of how much a food raises an individual’s blood glucose levels. Recent randomized controlled trials have found that a high glycemic load diet can increase the risk for acne [2-4]. The Dogon diet includes foods with low glycemic loads such as peanuts (GL: 1.7), mangoes (GL: 6.8), and millet (GL: 16.8). [1,17 (Power 2000?] These contrast sharply with the glycemic load of a traditional western diet that is high in refined sugar (GL: ~64.9) and white bread (GL: ~34.7) [1,17]. The Dogon do not have access to sweet deserts; however, they do tend to put a lot of sugar in their tea, coffee, and drinkable millet porridge. Nonetheless, their staple food (solid millet tôh) is not processed and has no sugar added.

**Milk.** The Dogon’s limited consumption of milk may also decrease their risk for acne. Consumption of cow’s milk increases insulin-like growth factors (e.g., IGF-1), which stimulate the sebaceous glands to secrete sebum and the adrenal glands to synthesize androgens [18] both of which are critical to the pathogenesis of acne. In many countries, milk is promoted for consumption and has been found to exacerbate acne development [19,20].

**PUFAs (polyunsaturated fatty acids).** Another possible reason for the Dogon’s low severity and prevalence of acne is their use of oil from the shea plant (Vitellaria paradoxa), which is rich is omega-3 fatty acids. High ratios of omega-6 PUFAs (in comparison to omega-3 PUFAs) promote inflammation, one of the processes involved in the development of acne [8,21,22]. Foods with high ratios of omega-6 to omega-3 are often found in the processed foods of modern lifestyles. Despite the Dogon’s traditional use of shea oil, they increasingly use palm oil, which has a higher ratio of omega-6 fatty acids. The use of palm oil may contribute to acne in the Dogon.

**PCBs and Other Pollutants.** Another reason for the Dogon’s low prevalence of acne may be limited exposure to halogenated pollutants, a type of pollutant common in industrial communities [23]. For example, chloracne is a type of acne caused by exposure to halogenated aromatic hydrocarbons such as PCBs and other polychlorinated compounds [9]. Most cases of chloracne occur from exposure to these pollutants during chemical manufacturing or exposure via contaminated industrial waste and contaminated food products [9]. The Dogon’s rural location, with limited contact to large-scale manufacturing, may help to explain their lower levels of acne prevalence and severity. Also, The Dogon consume very few canned foods, so they are not exposed to PCBs that way either, but it is unknown what level of exposure they might be getting from the plastic bags and plastic water containers that are ubiquitous in Dogon villages.

**Chronic Stress.** Stress, which exists in all human populations, has been shown to augment acne levels by causing an increase in glucocorticoids [24] which promote acne development [10]. For example, the total number of acne lesions [24] and the total number of free fatty acids (FFA) (an indication of the level of acne pathogenesis) increased significantly in response to stressful academic testing (Kraus 1970). Thus, stress is another factor to consider when analyzing acne levels for an individual or a community.

**Inflammation & Diseases of Modern Lifestyles.**  In evaluating the hypothesis that acne is a disease of modern lifestyles, it is helpful to consider inflammation. Inflammation plays an important role in the development of acne [25] and in the etiology of other chronic diseases often associated with a modern lifestyle (e.g., cardiovascular disease, type 2 diabetes mellitus, Alzheimer’s disease, and various cancers) [22]. For example, inflammation is promoted by both a high glycemic load diet and a high ratio of omega-6 to omega-3 PUFAs [22], both of which have also been found to exacerbate acne [4] and other chronic diseases [22]. The similar etiologies of acne vulgaris and other more traditional chronic diseases supports the hypothesis that acne vulgaris (especially moderate to severe inflammatory acne) is associated with modern lifestyles.

**SI REFERENCES**

1. Cordain L, Lindeberg S, Hurtado M *et al.* Acne vulgaris: a disease of Western Civilization. *Arch Dermatol* 2002;**38**:1584-90.

2. Smith RN, Braue A, Varigos GA *et al.* The effect of a low glycemic load diet on acne vulgaris and the fatty acid composition of skin, surface triglyceride *J Dermatol Sci* 2005;**50**:41-52.

3. Smith RN, Mann NJ, Braue A *et al.* A low glycemic-load diet improves symptoms in acne vulgaris patients: a randomized controlled trial. *Am J Clin Nutr* 2007;**86**:107-15.

4. Smith RN, Mann N, Makelainen H *et al.* A pilot study to determine the short-term effects of a low glycemic load diet on hormonal markers of acne: a nonrandomized, parallel controlled feeding trail. *Mol Nutr Food Res* 2008;**52**:718-26.

5. Ingram JR, Grindlay JC, Williams HC. Management of acne vulgaris: an evidence-based update. *Clinical and Experimental Dermatology* 2009;**35**:351-54.

6. Bowe W, Joshi S, Shalita A. Diet and acne. *American Academy of Dermatology*. 2009;**63**:124-41.

7. Adebamowo CA, Spiegelman D, Danby FW *et al.* High school dietary dairy intake and teenage acne *J Am Acad Dermatol* 2005;**52**:207-14.

8. Simopoulos AP. Omega-3 fatty acids in inflammation and autoimmune diseases *J Am Coll Nutr* 2002;**21**:495-505.

9. English JSC, Dawe RS and Ferguson J. Environmental effects and skin disease. *British Medical Bulletin* 2003;**68**:129-42. doi: 10.1093/bmb/ldg026.

10. Arora M, Yadav A, Saini V. Role of hormones in acne vulgaris. *J Clin Biochem* 2011;**44**:1035-40.

11. Danby W. Nutrition and acne. *Clinics in Dermatology* 2010;**28**:598-604.

12. Kaymak Y, Adisen E, Itler, N *et al.* Dietary glycemic index and glucose, insulin, insulin- like growth factor-I, insulin-like growth factor binding protein 3, and leptin levels in patients with acne. *J Am Acad Dermatol* 2007; **57**:819-23.

13. Berra B, Rizzo A. Glycemic Index, Glycemic Load: New Evidence for a Link with Acne. *Journal of the American College of Nutrition* 2009; **28**(suppl):450S-54S.

14. Holt SH, Miller JC, Petocz P. An insulin index of foods: the insulin demand generated by 1000-kJ portions of common foods. *Am J Clin Nutr* 1997;**66**:1264-76.

15. Burkhart CN, Gottwalk L. Assessment of etiologic agents in acne pathogenesis. *Skin Med* 2003;**2**:222-28.

16. Degitz K, Placzek M, Borelli C *et al.* Pathophysiology of acne. *JDDG* 2007;**4**:316-23. doi: 10.1111/j. 1610-0387.

17. Foster-Powell K, Holt S, Brand-Miller J. International table of glycemic index and glycemic load values: 2002. *Am J Clin Nutr* 2002; **76**:5-56.

18. Melnik B. Milk Consumption: aggravating factor of acne and promoter of chronic diseases in Western societies. *JDDG* 2009;**4**:364-70. doi: 10.1111/j.1610-0387.2009.07019.

19. Adebamowo CA, Spiegelman D, Berkey CS et al. Milk consumption and acne in adolescent girls. *Dermatology Online Journal* 2006;**12**. http://escholarship.org/uc/item/77b9s0z8

20. Adebamowo CA. Milk consumption and acne in teenage boys. *J Am Acad Dermatol* 2008;**58**:787-793. doi: 10.1016/j.jaad.2007.08.049.

21. Logan A. Dietary fat, fiber and acne vulgaris. *J Am Acad Dermatol* 2007;**57**:1092-93.

22. Galland L. Diet and Inflammation. *Nutrition in Clinical Practice* 2010;**25**:634-40.

23. Ju Q, Zouboulis C, Xia L. Environmental pollution and acne: Chloracne. *Dermato-Endocrinology* 2009;**1**:125-28.

24. Kapes B. Stress Linked to Acne Severity. *Dermatology Times* 2003; **24**:19.

25. Harvey A, Huynh T. Inflammation and Acne: Putting the Pieces Together. *Journal of Drugs in Dermatology*, 2014;**13**:459-463.
